# Plasma Anion Gap and Risk of In-Hospital Mortality in Patients with Acute Ischemic Stroke: Analysis from the MIMIC-IV Database

**DOI:** 10.3390/jpm11101004

**Published:** 2021-10-04

**Authors:** Hong-Jie Jhou, Po-Huang Chen, Li-Yu Yang, Shu-Hao Chang, Cho-Hao Lee

**Affiliations:** 1 Department of Neurology, Changhua Christian Hospital, Changhua City 500, Taiwan; xsai4295@gmail.com (H.-J.J.); vicky102433@gmail.com (L.-Y.Y.); 2 School of Medicine, Kaohsiung Medical University, Kaohsiung 807, Taiwan; 3 Department of Internal Medicine, Tri-Service General Hospital, National Defense Medical Center, Taipei 114, Taiwan; chenpohuang@hotmail.com; 4 Department of Computer Science and Information Science, National Formosa University, Yunlin 632, Taiwan; skyheero@gmail.com; 5 Division of Hematology and Oncology Medicine, Department of Internal Medicine, Tri-Service General Hospital, National Defense Medical Center, Taipei 114, Taiwan

**Keywords:** ischemic stroke, anion gap, intensive care unit, MIMIC-IV

## Abstract

We aimed to investigate the association between the plasma anion gap (AG) and in-hospital mortality among patients with acute ischemic stroke (AIS). In total, 1236 AIS patients were enrolled using the Medical Information Mart for Intensive Care Database IV. Primary outcome was in-hospital mortality. The patients were divided into four groups according to AG category. The mean age and Charlson comorbidity index increased as the AG category increased. The fourth AG category was most related to the in-hospital mortality (hazards ratio (HR), 95% confidence interval (CI): 2.77, 1.60–4.71), even after adjusting for possible confounding variables (Model 1: HR, 95% CI: 3.37, 1.81–6.09; Model 2: HR, 95% CI: 3.57, 1.91–6.69). Moreover, intensive care unit mortality (*p* = 0.008) was higher in the highest AG category, but the intracranial hemorrhage (*p* = 0.071) did not associate with the plasma AG. The plasma AG had a satisfactory predictive ability for in-hospital mortality among AIS patients (areas under the receiver operating characteristic curve: 0.631). The plasma AG is an independent risk factor that can satisfactorily predict the in-hospital mortality among AIS patients.

## 1. Introduction

According to the report of the Global Burden of Disease Study 2017, stroke is the second leading cause of death and disability worldwide. The increased burden of stroke puts tremendous pressure on patients, their families, and society [1]. Previous studies have mainly focused on the survival and functional outcome of stroke patients in the general population [2]. However, because of an aging society and the promising reperfusion therapy, multiple interventions and the intensive care might be necessary for these patients. Thus, early identification of patients with the highest risk of adverse outcomes is of considerable importance regarding both the prognostication and targeting of appropriate therapies. To be considered useful, risk markers should be readily available, provide incremental information, and possess a clear pathophysiological basis.

The plasma anion gap (AG) is commonly used to classify acid–base disorders and to diagnose various conditions. It is easily calculated by subtracting the plasma concentration of anions (chloride and bicarbonate) from that of cations (sodium). Previous studies have demonstrated that the plasma AG might be associated with mortality in patients with acute kidney injury, acute myocardial infarction, congestive heart failure, acute pancreatitis, and aortic aneurysm [3,4,5,6,7]. The changes in blood gases and electrolytes are some of the first biochemical responses to ischemic stroke [8]. Thus, they have the potential to be used as predictors of stroke outcomes [8]. In previous studies by Liu et al., results show that a high AG was associated with an increased risk of all-cause mortality in critically ill patients with cerebral infarction [9]. However, the anion gap is not often reliable to identify increased concentrations of gap anions. Particularly, hypoalbuminemia, a common disturbance in critically ill patients, might result in underestimation of the value of the anion gap.

Thus, this study aims to investigate whether the initial plasma AG is an appropriate predictor for in-hospital mortality, intensive care unit mortality, and incidence of intracerebral hemorrhage to stratify the severity of illness in patients with ischemic stroke for the purpose of quality assurance or not.

## 2. Materials and Methods

### 2.1. Study Population and Data Source

This is a retrospective study using the Medical Information Mart for Intensive Care (MIMIC)-IV database (version: 1.0) [10]. This database, an update to MIMIC-III, is deidentified according to the Health Insurance Portability and Accountability Act Safe Harbor provision and has an approval from Massachusetts Institute of Technology and Institutional Review Board of Beth Israel Deaconess Medical Center (BIDMC) [11]. The MIMIC-IV contains clinical information of patients in the intensive care unit (ICU) at BIDMC between 2008 and 2019. One author, Hong-Jie Jhou, has finished the Collaborative Institutional Training Initiative examination (certification number: 39050603) and achieved access to the database for data extraction.

### 2.2. Study Population and Variable Extraction

**Figure 1:**
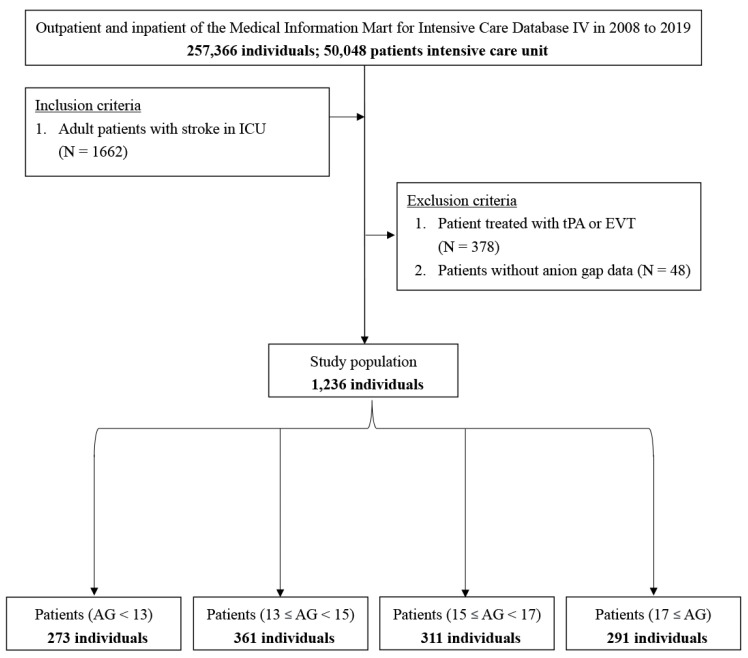
Flowchart of study patients.

Patients from 2008 to 2019 were identified in the MIMIC-IV database. The inclusion criteria were as follows: adult patients (age, 18–89 years) with ischemic stroke, defined as ICD-9 codes of 433, 434, 436, 437.0, and 437.1 or ICD-10 codes of I63, I65, and I66 (Figure 1). We excluded patients who received acute reperfusion therapy, such as intravenous tissue plasminogen activator or endovascular mechanical thrombectomy. Patients who had a history of transient ischemic attack without deterioration into ischemic cerebral infarction and those who lacked data of interest, such as the AG value during the hospitalization, were also excluded. We adopted the date of the first ICU admission only for patients who were admitted to the ICU more than once.

The patient characteristics were collected as follows: (1) comorbidities: hypertension, hyperlipidemia, diabetes mellitus, coronary artery disease, congestive heart failure, peripheral vascular disease, chronic obstructive pulmonary disease, liver disease, peptic ulcer disease, chronic kidney disease, rheumatoid arthritis, dementia, malignancy, atrial fibrillation; (2) severity scoring system: systemic inflammatory response syndrome (SIRS) score, sequential organ failure assessment (SOFA) score, Simplified Acute Physiology Score (APS) III [12], and HAS-BLED score [13] (Table S1); (3) the first value of vital signs and laboratory data, within 24 h of ICU admission. Eighteen categories of medical conditions were identifiable in the medical records for the overall Charlson comorbidity index [14,15] (CCI) (Table S2). Secondary prevention medication for ischemic stroke were identified, including antiplatelet agents (e.g., aspirin, clopidogrel, ticlopidine, cilostazol, ticagrelor, prasugrel, and dipyridamole) and anticoagulation agents (e.g., warfarin, dabigatran, apixaban, rivaroxaban, and edoxaban) [16].

### 2.3. Definition of AG and Outcome Measurement

AG was defined using the following equation: AG (initial, [mmol/L]) = plasma sodium [mmol/L] − (plasma chloride [mmol/L] + plasma total bicarbonate [mmol/L]). Corrected AG was calculated using the equation of Figge–Jabor–Kazda–Fencl: corrected AG = observed AG + 0.25 × (normal albumin−observed albumin) [17]. The primary outcome was set as the in-hospital mortality. The secondary outcome was ICU mortality as well as incidence of intracerebral hemorrhage. Survival information was extracted from the table named “patients” of the MIMIC-IV database. Data regarding the length of hospital stay were extracted from the table named “admissions” of the MIMIC-IV database [11].

### 2.4. Statistical Analysis

Categorical variables were represented as numbers (percentages) and were compared using the Chi-square and Fisher exact tests. Continuous variables were described as means (standard deviation) and were compared using the Kruskal–Wallis test or one-way analysis of variance.

**Figure 2:**
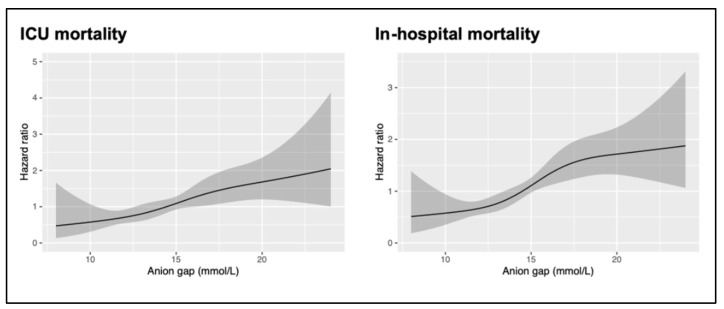
Relationship between the plasma AG and the risk of ICU and in-hospital mortality. Shaded areas around the curves depict 95% confidence intervals. AG: anion gap; ICU: intensive care unit.

We measured the linearity assessment of the AG value with the ICU and in-hospital mortality of patients with ischemic stroke with a restricted cubic splines model. The restricted cubic spline curve and univariate Cox-proportional hazards regression of the ICU and in-hospital mortality showed approximate linear regression (Figure 2). The patients were classified into four groups on the basis of four AG categories (AG < 13 mmol/L, 13 mmol/L ≤ AG < 15 mmol/L, 15 mmol/L ≤ AG < 17 mmol/L, and AG ≥ 17 mmol/L). The separated groups were compared using the log-rank test, and the Kaplan–Meier method was used to estimate the absolute risk of each event for each group.

Univariate and multivariate Cox hazards model analyses were performed to identify the association between the AG and ICU and in-hospital mortality. In model 1, the covariates were adjusted only for those that were unequal in the baseline characteristics. Model 2 included the adjusted variables, including model 1, and the clinically relevant factors such as vital signs and laboratory data. The results were expressed as the hazards ratio (HR) with a 95% confidence interval (CI). The risk factors for multivariate adjustment were selected as potential covariates for a poor outcome after ischemic stroke on the basis of prior knowledge. The logistic regression of the incidence of intracerebral hemorrhage was applied with the adjustments of models 1 and 2. The receiver operating characteristic (ROC) analysis was further drawn to evaluate the ability of the SIRS score, SOFA score, and AG to predict in-hospital mortality.

All comparisons were planned, the tests were two-sided, and *p*-values of less than 0.05 were used to denote statistical significance between two or more groups. Statistical analyses were performed using the MedCalc Statistical Software version 20.011 (MedCalc Software, Ostend, Belgium), Statistical Package for the Social Sciences (SPSS, version 25.0; IBM Corp., Armonk, NY, USA), and R Version 4.0.1 (R Core Team (2020), R Foundation for Statistical Computing, Vienna, Austria).

## 3. Results

### 3.1. Patient Characteristics

A total of 257,366 medical records were reviewed, and 50,048 patients were admitted to the ICU. We excluded 378 patients receiving tissue plasminogen activator or endovascular mechanical thrombectomy and 48 patients without the value of anion gap. In total, 1236 patients with ischemic stroke were included in the study (Figure 1). Table 1 summarizes the basic demographic characteristics of the patients stratified by their AG. The patients were aged 68.7 ± 14.2 years and comprised 577 (46.7%) females. According to the AG, 273, 361, 311, and 291 patients belonged to the first (<13 mmol/L), second (≥13 and <15 mmol/L), third (≥15 and <17 mmol/L), and fourth (≥17  mmol/L) category, respectively. The patients with AG ≥ 17 mmol/L were older, had a higher CCI and APS III, and had more comorbidities such as hyperlipidemia, congestive heart failure, diabetes mellitus, chronic kidney disease, and atrial fibrillation (Table 1).

### 3.2. Association between AG and Outcomes

**Figure 3:**
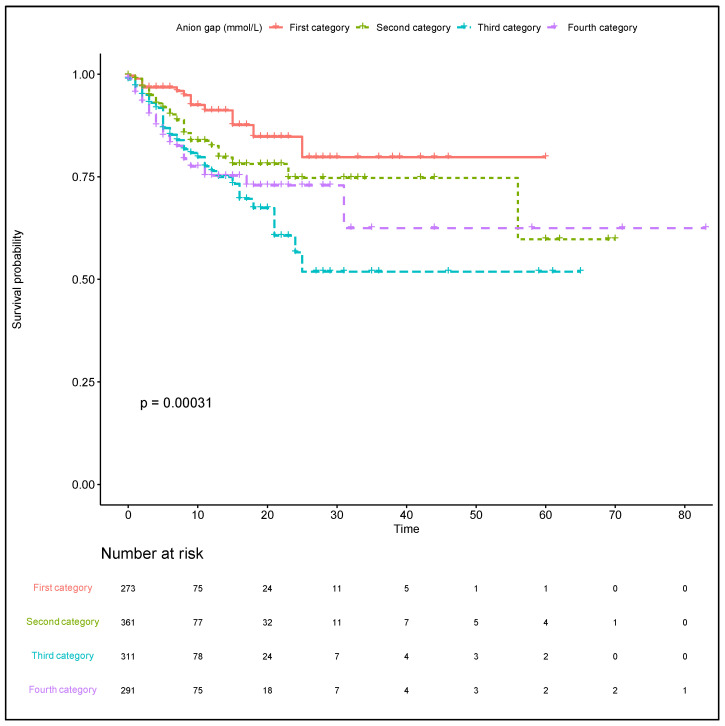
Kaplan–Meier curves indicating the association between the AG category and in-hospital mortality. Red line: first category. Green line: second category. Blue line: third category. Purple line: fourth category. AG: anion gap.

The first category AG was used as a baseline reference for comparison with other category groups in the association analyses (Table 2). In the original cohort using univariate Cox regression analysis, the highest AG (fourth category vs. first category) was related to higher risk of in-hospital mortality (crude HR, 2.77; 95% CI, 1.60–4.79; *p* < 0.001). In model 1, the highest AG was associated with an increased risk of in-hospital mortality after adjusting for age, sex, vital signs, and comorbidities (adjusted HR, 3.37; 95% CI, 2.16–6.09; *p* < 0.001). In model 2, after adjustments for model 1 and the additional clinically relevant factors, a highest AG remained significantly associated with an increase in in-hospital mortality rates (adjusted HR, 3.57; 95% CI, 1.91–6.69; *p* < 0.001). We had corrected the influence of hypoalbuminemia among critically ill patients for the AG, and a positive trend was also noted ICU mortality (*p* = 0.0046; Figure S1A) and in-hospital mortality (*p* = 0.0006; Figure S1B). The similar results were showed in ICU mortality; however, the AG was not associated with incidence of intracerebral hemorrhage (Table 2). The Kaplan–Meier curves demonstrated the association between the AG category and the in-hospital mortality (Log-rank test *p*-value: 0.00031; Figure 3). The comparison of Kaplan–Meier estimate between each two groups was summarized in Table S3. Survival was followed until hospital discharge, and the longest length of hospital stay was 83 days.

### 3.3. ROC Curve Analysis

**Figure 4:**
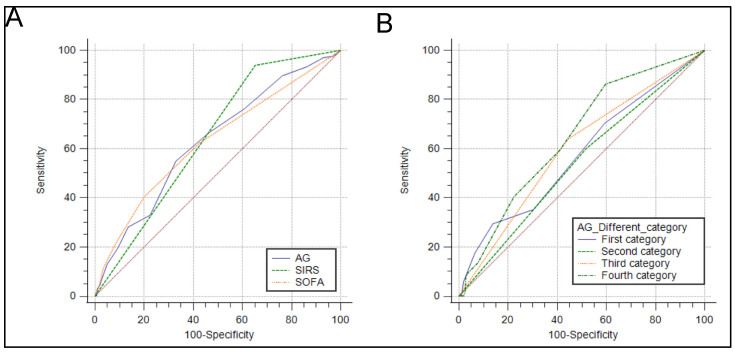
Receiver operating characteristic curves for the prediction of the in-hospital mortality in critically ill patients with ischemic stroke. (**A**) The ability of the SIRS score, SOFA score, and AG to predict in-hospital mortality. (**B**) The ability of different AG category to predict in-hospital mortality. AG: anion gap; SIRS: systemic inflammatory response syndrome; SOFA: sequential organ failure assessment score.

The SIRS and SOFA scores were a scoring tool that provided a potential prediction of in-hospital mortality. The sensitivity and specificity of the AG and SIRS and SOFA scores were tested using ROC curves. Meanwhile, to evaluate the predictive performance of the AG for the in-hospital mortality, the area under the ROC curve (AUC) was calculated. The AUC was 0.631 (95% CI: 0.603–0.658) for the AG, 0.644 (95% CI: 0.616–0.670) for the SIRS score, and 0.628 (95% CI: 0.600–0.655) for the SOFA score (Figure 4A). The predictive abilities based on the AG category were evaluated using ROC curves (AUC, 0.536; 95% CI, 0.475–0.597; AUC, 0.532; 95% CI, 0.478–0.584; AUC, 0.609; 95% CI, 0.552–0.663; and AUC, 0.644; 95% CI, 0.585–0.698, from the first to fourth category, respectively; Figure 4B). Furthermore, after adjustment according to the equation of Figge, the AUC of the corrected AG was 0.683 (95% CI, 0.641–0.723; Figure S2).

## 4. Discussion

This study is to clearly reveal the potential linear trend between the plasma AG and in-hospital mortalities. This large retrospective cohort study of critically ill patients with acute ischemic stroke has demonstrated that patients with an elevated plasma AG are more likely to have poor clinical outcomes and a higher risk of in-hospital mortality, even after adjustments for traditional cerebrovascular risk factors. Although the plasma AG may improve the discrimination and reclassification of patients at risk, the predictive ability of AG regarding the risk of in-hospital mortality is only satisfactory, even in the model that corrected the influence of hypoalbuminemia. However, an inexpensive clinical indicator can be readily used by physicians to evaluate the prognosis among stroke patients.

Stroke remains one of the leading causes of death and disability worldwide, and there are presently no appropriate prognostic biomarkers for physicians early in the course of the disease. Recently, several studies have focused on the clinical value of the plasma AG, which is obtained from the concentration of plasma sodium, chloride, and bicarbonate, to predict the risk in critically ill patients. In a retrospective study that analyzed 6868 hospitalized patients, Lolekha et al. [18] showed that nearly 40.5% of patients have an abnormal plasma AG, which included 37.6% with a high plasma AG and 2.9% with a low plasma AG. Compared with traditional biomarkers such as blood gas analysis or lactate, the plasma AG is inexpensive and readily available in low-resource settings [19].

Data regarding the association between the plasma AG and ischemic stroke are scarce; however, some evidence shows that it is, at least, partially causal [9]. An increased plasma AG usually indicates an imbalance between acid generation caused by tissue hypoperfusion and acid excretion based on the renal function [20]. Thus, the presence of an increased plasma AG may reflect subtle hemodynamic abnormalities caused by tissue hypoperfusion but not overt shock [20]. Additionally, tissue acidosis has been shown to have deleterious effects on ischemic injury in the nervous system [21]. At the cellular level, acidotoxic cellular calcium accumulation and cytotoxic edema, which are mediated by ion channels sensitive to an acidic shift in tissue pH (e.g., the acid-sensing ion channel 1a, proton-activated chloride channels, and sodium-hydrogen exchanger isoform 1), may be limited with the use of selective ion channel blockers. This leads to spreading depolarization with the evolution of ischemic stroke, which might aggravate tissue acidosis and induce cell death [22]. In a previous cohort study, the plasma AG was independently associated with the risk of mortality [9]. In this study, we expounded on that finding, revealing that there was a positive linear trend between the plasma AG and the in-hospital mortality.

Blood gas analysis, as an alternative means to evaluate acid–base disturbances, might also predict prognosis in critically ill patients; however, blood gas analysis can be influenced by a compensatory respiratory alkalosis. The plasma AG is relatively independent of acute respiratory changes and a sensitive tool of metabolic derangement [4]. Furthermore, the calculation of the plasma AG is simple and does not require an arterial puncture. In this study, the plasma AG was an independent predictor for in-hospital mortality in patients with ischemic stroke. We noted that the level of pH was not associated with the in-hospital mortality. Furthermore, hypoalbuminemia was commonly seen in critically ill patients, which might result in the underestimation of the AG. We adjusted the AG by using the equation of Figge [17,23]. The results showed that an elevated corrected AG significantly increased the risk of in-hospital mortality. Thus, the plasma AG may be a viable option to evaluate the prognosis among critically ill patients with ischemic stroke.

In a meta-analysis, Glasmacher et al. [19] included 19 studies to estimate the accuracy of the AG in critically ill patients. The summary AUC of plasma AG was 0.72 with 95% CI 0.59–0.86, and the summary AUC of the corrected AG was 0.67 with 95% CI 0.62–0.71. However, high statistical heterogeneity was found in the meta-analyses. Similarly, in our study, the plasma AG only presented a satisfactory prediction ability for in-hospital mortality (AUC was 0.631 with 95% CI 0.603–0.658). The highest prediction rate (AUC was 0.644 with 95% CI 0.585–0.698) was noted when the plasma AG was ≥17 mmol/L. Consequently, using a single measurement of plasma AG may not be an effective tool for risk classification. Hence, a tailored evaluation strategy should be considered for patients with ischemic stroke.

The main strength of our study was being a large and diverse population study design using real-world data. However, the results should be elucidated in the context of the following limitations. First, this was a retrospective study, and the diagnosis of ischemic stroke was according to the administrative diagnosis codes. Although the first sequence of diagnosis was used in this study, there remained the possibility of misclassifications that could cause false associations. Second, hypoalbuminemia was commonly seen in critically ill patients, which might result in the underestimation of the AG. Thus, we used the equation of Figge to correct the AG, and the results showed a similar trend for the in-hospital mortality. Third, because of the nature of the MIMIC database, we lacked some potential variables such as the National Institute of Health Stroke Scale and the subtypes of ischemic stroke (TOAST classification). Therefore, we used other severity scoring systems such as the SIRS and SOFA scores as well as the simplified acute physiology score III to assess the severity of stroke. No long-term follow-up events were provided from the MIMIC-IV database, so the functional outcomes and poststroke disposition of patients were unknown. Finally, the study might have selection bias, as it was a single-institution study. Some stroke patients might not be referred from other hospitals because of a narrow window time for reperfusion therapy after symptom onset of ischemic stroke and the evolution of stroke severity. These high-risk patients might not have been enrolled in our study. Furthermore, the patients with a low severity of ischemic stroke might be admitted to the general ward and not involved in our analysis. Hence, to investigate the external generalizability, further studies are necessary.

## 5. Conclusions

The AG was an independent risk factor for in-hospital mortality and was associated with adverse clinical outcomes among ischemic stroke patients. According to our results, an AG of ≥17 mmol/L had a satisfactory ability to predict a poor prognosis. However, to confirm the AG’s role as a clinical indicator for the prognosis of ischemic stroke patients, more prospective case-control studies are warranted.

## Figures and Tables

**Table 1:** Characteristics of the study patients.

	Anion Gap (mmol/L)				
Characteristics	Category 1 (*n* = 273)	Category 2 (*n* = 361)	Category 3 (*n* = 311)	Category 4 (*n* = 291)	*p*-Value
Age (years)	68.30 ± 13.26	68.70 ± 14.20	68.14 ± 14.94	69.61 ± 14.39	0.595
Gender, *n*					0.134
Male	158 (57.9%)	200 (55.4%)	155 (49.8%)	146 (50.2%)	
Female	115 (42.1%)	161 (44.6%)	156 (50.2%)	145 (49.8%)	
Race, *n*					0.027
White	187 (68.5%)	242 (67.0%)	203 (65.3%)	188 (64.6%)	
Black	24 (8.8%)	36 (10.0%)	21 (6.8%)	36 (12.4%)	
Asian	6 (2.2%)	17 (4.7%)	4 (1.3%)	5 (1.7%)	
Other	56 (20.5%)	66 (18.3%)	83 (26.6%)	62 (21.3%)	
MAP (mmHg)	92.34 ± 19.41	94.84 ± 17.21	96.62 ± 16.84	96.68 ± 18.83	0.013
Temperature (°C)	36.71 ± 0.52	36.80 ± 0.61	36.80 ± 0.50	36.85 ± 0.58	0.043
Heart rate (beats/minute)	76.95 ± 17.10	80.05 ± 17.09	81.36 ± 17.61	86.66 ± 17.95	<0.001
Respiratory rate (breath/minute)	18.37 ± 4.83	18.29 ± 4.79	18.63 ± 4.97	19.70 ± 5.62	0.002
SpO2 (%)	96.98 ± 3.78	96.92 ± 2.75	97.30 ± 2.62	96.84 ± 3.13	0.271
Comorbidities, *n*					
CCI	6.64 ± 2.54	6.64 ± 2.57	6.67 ± 2.54	7.35 ± 2.89	0.001
Hypertension	192 (70.3%)	264 (73.1%)	230 (74.0%)	217 (74.6%)	0.683
Hyperlipidemia	78 (28.6%)	175 (20.8%)	170 (22.5%)	57 (19.6%)	0.052
Diabetes mellitus	83 (30.4%)	111 (30.7%)	100 (32.2%)	118 (40.5%)	0.027
Coronary artery disease	34 (12.5%)	43 (11.9%)	36 (11.6%)	43 (14.8%)	0.636
Congestive heart failure	50 (18.3%)	51 (14.1%)	49 (15.8%)	75 (25.8%)	<0.001
PVD	39 (14.3%)	40 (11.1%)	42 (13.5%)	26 (8.9%)	0.178
COPD	51 (18.7%)	66 (18.3%)	52 (16.7%)	49 (16.8%)	0.893
Liver disease					
Mild	8 (2.9%)	7 (1.9%)	13 (4.2%)	8 (2.7%)	0.391
Moderate to severe ^#^	0 (0.0%)	1 (0.3%)	4 (1.3%)	2 (0.7%)	0.178 ^#^
Peptic ulcer disease ^#^	4 (1.5%)	5 (1.4%)	4 (1.3%)	1 (0.3%)	0.509 ^#^
Chronic kidney disease	30 (11.0%)	43 (11.9%)	48 (15.4%)	60 (20.6%)	0.004
Rheumatoid arthritis	5 (1.8%)	14 (3.9%)	5 (1.6%)	6 (2.1%)	0.198
Dementia	13 (4.8%)	16 (4.4%)	14 (4.5%)	13 (4.5%)	0.997
Malignancy	22 (8.1%)	22 (6.1%)	21 (6.8%)	19 (6.5%)	0.801
Atrial fibrillation	82 (30.0%)	116 (32.1%)	118 (37.9%)	124 (42.6%)	0.006
Laboratory parameters					
WBC (10^9^/L)	9.12 ± 4.73	9.75 ± 4.15	10.67 ± 4.36	11.29 ± 4.84	<0.001
Hgb (g/dL)	11.92 ± 2.07	12.41 ± 2.15	12.62 ± 2.12	12.65 ± 2.11	<0.001
Platelet (10^9^/L)	211.10 ± 79.14	229.31 ± 82.09	237.78 ± 105.29	243.77 ± 97.58	<0.001
Creatinine (mEq/L)	0.90 ± 0.34	0.98 ± 0.48	1.03 ± 0.60	1.39 ± 1.65	<0.001
BUN (mg/dL)	17.06 ± 8.39	17.37 ± 9.80	19.13 ± 9.59	23.29 ± 17.83	<0.001
Sodium (mmol/L)	139.37 ± 4.01	139.35 ± 3.60	139.71 ± 4.13	139.17 ± 4.15	0.404
Potassium (mmol/L)	4.01 ± 0.52	4.05 ± 0.55	4.02 ± 0.60	4.16 ± 0.65	0.011
Bilirubin (mg/dL)	0.58 ± 0.46	0.62 ± 0.46	0.62 ± 0.49	0.69 ± 0.59	0.272
pH level	7.41 ± 0.08	7.39 ± 0.07	7.39 ± 0.09	7.39 ± 0.08	0.286
Drugs, *n*					
Antiplatelet agents	238 (87.2%)	296 (82.0%)	244 (78.5%)	226 (77.7%)	0.015
Anticoagulation agents					
Warfarin	58 (21.2%)	82 (22.7%)	74 (23.8%)	70 (24.1%)	0.853
NOAC	15 (5.5%)	16 (4.4%)	11 (3.5%)	26 (8.9%)	0.021
HAS-BLED score	3.60 ± 0.97	3.68 ± 0.89	3.72 ± 0.93	3.83 ± 0.94	0.025
APS III	36.59 ± 17.33	36.23 ± 18.07	40.12 ± 19.67	43.40 ± 21.81	<0.001
SIRS score	1.88 ± 0.99	1.93 ± 0.98	2.19 ± 0.99	2.17 ± 0.98	<0.001
SOFA score	0.81 ± 1.21	0.79 ± 1.16	0.76 ± 1.28	1.09 ± 1.44	0.005
ICU mortality, *n*	11 (4.0%)	23 (6.4%)	32 (10.3%)	33 (11.3%)	0.003
ICU length of stay, day	4.26 ± 5.46	4.03 ± 4.81	4.48 ± 5.44	3.94 ± 4.13	0.533
In-hospital mortality, *n*	17 (6.2%)	40 (11.1%)	55 (17.7%)	52 (17.9%)	<0.001
Hospital length of stay, day	8.02 ± 9.26	7.81 ± 9.58	8.00 ± 8.62	8.11 ± 9.25	0.979
Intracranial hemorrhage, *n*	22 (8.1%)	27 (7.5%)	40 (12.9%)	33 (11.3%)	0.067
PEG/PEJ tube placement, *n*	33 (12.1%)	38 (10.5%)	34 (10.9%)	34 (11.7%)	0.926

APS III: acute physiology score III; BUN: blood urea nitrogen; CCI: Charlson comorbidity Index; COPD: chronic obstructive pulmonary disease; Hgb: hemoglobin; MAP: mean arterial pressure; NOAC: novel oral anticoagulant; PVD peripheral vascular disease; SIRS: systemic inflammatory response syndrome; SpO2: saturation of peripheral oxygen; SOFA: sequential organ failure assessment score; PEG: percutaneous endoscopic gastrostomy; PEJ: percutaneous endoscopic jejunostomy; WBC: white blood cell. #: Testing by Fisher exact test, or Kruskal–Wallis test, respectively.

**Table 2:** Association between different anion gap levels and outcomes among stroke patients.

	Cohort—Univariate		Model 1—Multivariate		Model 2—Multivariate	
Outcomes	Crude HR (95% CI)	*p* Value	Adjusted HR (95% CI)	*p* Value	Adjusted HR (95% CI)	*p* Value
ICU Mortality	*n* = 1236	0.008	*n* = 1229	<0.001	*n* = 11,217	<0.001
Category 1	Reference		Reference		Reference	
Category 2	1.58 (0.76–3.26)	0.220	2.20 (1.01–4.79)	0.048	2.36 (1.06–5.23)	0.035
Category 3	2.40 (1.21–4.76)	0.012	3.47 (1.67–7.18)	0.001	3.72 (1.76–7.88)	0.001
Category 4	2.91 (1.47–5.77)	0.002	4.52 (2.16–9.48)	<0.001	5.51 (2.47–12.30)	<0.001
In-hospital Mortality	*n* = 1236	0.001	*n* = 1229	<0.001	*n* = 1217	<0.001
Category 1	Reference		Reference		Reference	
Category 2	1.80 (1.02–3.18)	0.042	2.26 (1.25–4.07)	0.007	2.41 (1.31–4.43)	0.005
Category 3	2.75 (1.59–4.73)	<0.001	3.21 (1.81–5.72)	<0.001	3.19 (1.77–5.76)	<0.001
Category 4	2.77 (1.60–4.79)	<0.001	3.37 (1.86–6.09)	<0.001	3.57 (1.91–6.69)	<0.001
Intracerebral Hemorrhage	*n* = 1236	0.071	*n* = 1229	0.290	*n* = 1217	0.630
Category 1	Reference		Reference		Reference	
Category 2	0.92 (0.51–1.66)	0.787	1.08 (0.55–2.12)	0.828	1.03 (0.52–2.06)	0.922
Category 3	1.68 (0.97–2.91)	0.062	1.70 (0.89–3.24)	0.107	1.45 (0.75–2.82)	0.272
Category 4	1.46 (0.83–2.57)	0.191	1.46 (0.74–2.88)	0.269	1.19 (0.58–2.42)	0.634

HR: hazard ratio; OR: odds ratio. Model 1: All results of Adj-HR were adjusted by age, gender, race, heart rate, mean blood pressure, respiratory rate, temperature, Charlson comorbidity Index, hyperlipidemia, congestive heart failure, diabetes mellitus, chronic kidney disease, atrial fibrillation, HAS-BLED score, acute physiology score III, systemic inflammatory response syndrome, sequential organ failure assessment score, antiplatelet agents, anticoagulation agents (novel oral anticoagulant). Model 2: All result of Adj-HR were adjusted by age, gender, race, heart rate, mean blood pressure, respiratory rate, temperature, Charlson comorbidity Index, hyperlipidemia, congestive heart failure, diabetes mellitus, chronic kidney disease, atrial fibrillation, HAS-BLED score, acute physiology score III, systemic inflammatory response syndrome, quick sequential organ failure assessment score, antiplatelet agents, anticoagulation agents (novel oral anticoagulant), white blood cell, hemoglobin, platelet, potassium, blood urea nitrogen, creatinine.

## Data Availability

All data accessed and analyzed in this study are available in the article and its Supplementary Materials.

## Supplementary Materials

The following are available online at https://www.mdpi.com/article/10.3390/jpm11101004/s1, Figure S1: Relationship between the plasma corrected anion gap and the risk of intensive care unit and in-hospital mortality, Figure S2: Receiver operating characteristic curves for the predictive ability of plasma corrected anion gap for the in-hospital mortality in stroke patients, Table S1: ICD-9-CM and definition of HAS-BLED score, Table S2: ICD-9-CM and definition of Charlson comorbidity Index, Table S3: Comparison of Kaplan–Meier estimate.
