# Supplementary material for: Plasma Anion Gap and Risk of In-Hospital Mortality in Patients with Acute Ischemic Stroke: Analysis from the MIMIC-IV Database
Source: J Pers Med. 2021 Oct 4;11(10):1004. doi: 10.3390/jpm11101004 (PMC8541378; doi:10.3390/jpm11101004)
Supplement: Supplementary file 1 [file jpm-11-01004-s001.zip › jpm-1396484-supplementary.pdf]

Journal of
Personalized
Medicine

Supplementary Materials

Contents

Table S1: ICD-9-CM and definition of HAS-BLED score
Table S2: ICD-9-CM and definition of Charlson comorbidity Index
Table S3: Comparison of Kaplan–Meier estimate
Figure S1: Relationship between the plasma corrected AG and the risk of intensive care unit and in-hospital mortality
Figure S2: Receiver operating characteristic curves for the predictive ability of corrected anion gap for the in-hospital mortality in stroke patients

Table S1. - ICD-9-CM and definition of HAS-BLED score.

Item	ICD 9	ICD 10	score
Hypertension	401.1, 401.9, 402.10, 402.90, 404.10, 404.90, 405.11, 405.19, 405.91, 405.99	I10, I11, I12, I13, I15, N26.2	1
Renal disease	582, 585, 586, V56, 588.0, V42.0, V45.1, 403.01, 403.11, 403.91, 404.02, 404.03, 404.12, 404.13, 404.92, 404.93, 583.0, 583.1, 583.2, 583.3, 583.4, 583.5, 583.6, 583.7	N18, N19, I12.0, I13.1, N03.2, N03.3, N03.4, N03.5, N03.6, N03.7, N05.2, N05.3, N05.4, N05.5, N05.6, N05.7, N25.0, Z49.0, Z49.1, Z49.2, Z94.0, Z99.2	1
Liver disease	570, 571, 070.6, 070.9, 573.3, 573.4, 573.8, 573.9, V42.7, 070.22, 070.23, 070.32, 070.33, 070.44, 070.54, 456.0, 456.1, 456.2, 572.2, 572.3, 572.4, 572.8	B18, K73, K74, K70.0, K70.1, K70.2, K70.3, K70.9, K71.3, K71.4, K71.5, K71.7, K76.0, K76/2, K76/3, K76.4, K76.8, K76.9, Z94.4, I85.0, I85.9, I86.4, I98.2, K70.4, K71.1, K72.1, K72.9, K76.5, K76.6, K76.7	1
Stroke, transient ischemic attack	430, 431, 432.0, 432.1, 432.9, 433, 434, 436, 437.0, 437.1,	I60, I61, I62.0, I62.9, I63, I65, I66	1
Bleeding history	ICH: 430, 431, 432.0, 432.1, 432.9 Extra-cranial: 531.0, 531.2, 531.4, 531.6, 532.0, 532.2, 532.4, 532.6, 533.0, 533.2, 533.4, 533.6, 534.0, 534.2, 534.4, 534.6, 578.0, 578.1, 578.9, 569.3, 287.8, 287.9, 596.7, 784.8, 599.7, 627.1, 459.0, 719.1, 786.3	ICH: I60, I61, I62.0, I62.9 Extra-cranial: K92.0, K92.1, I85.0, I98.20, I98.3, K22.10, K22.12, K22.14, K22.16, K25.0, K25.2, K25.4, K25.6, K26.0, K26.2, K26.4, K26.6, K27.0, K27.2, K27.4, K27.6, K28.0, K28.2, K28.4, K28.6, K29.0, K63.80, K31.80, K55.20, K62.5, K92.2, N02.0, N02.1, N02.2, N02.3, N02.4, N02.5, N02.6, N02.7, N02.8, N02.9, K66.1, N93.8, N93.9, N95.0, R04.1, R04.2, R04.8, R04.9, R31.0, R31.1, R31.8, R58, D68.3, H35.6, H43.1, H45.0, M25.0	1
INR > 4	NA	NA	1

Age	NA	NA	1, age ≥ 65;<br>0, age < 65.
Medication usage predisposing to bleeding	NA	NA	1
Alcohol use	291.1, 291.2, 291.5, 291.8, 291.9, 303.93, 305.03, V11.3	F10, E52, G62.1, I42.6, K29.2, K70.0, K70.3, K70.9, T51, Z71.4, Z65.8	1

Table S2. - ICD-9-CM and definition of Charlson comorbidity Index.

Condition	ICD 9	ICD 10	Weight
Age score	NA	NA	4, Age ≥ 70;<br>3, 70 > Age ≥ 61;<br>2, 60 > Age ≥ 51;<br>1, 50 > Age ≥ 41;<br>0, Age < 40.
Myocardial infarct	410, 412	I21, I22, I25.2	1
Congestive heart failure	428, 398.91, 402.01, 402.11, 402.91, 404.01, 404.03, 404.11, 404.13, 404.91, 404.93, 425.4, 425.9	I43, I50, I09.9, I11.0, I13.0, I13.2, I25.5, I42.0, I42.5, I42.6, I42.7, I42.8, I42.9, P29.0	1
Peripheral vascular disease	440, 441, 093.0, 437.3, 447.1, 557.1, 557.9, V43.4, 443.1, 443.9	I70, I71, I73.1, I73.8, I73.9, I77.1, I79.0, I79.2, K55.1, K55.8, K55.9, Z95.8, Z95.9	1
Cerebrovascular disease	430, 431, 432, 433, 434, 435, 436, 437, 438, 362.34	G45, G46, I60~I69, H34.0	1
Dementia	290, 294.1, 331.2	F00, F01, F02, F03, G30, F051, G311	1
Chronic pulmonary disease	490, 491, 492, 493, 494, 495, 496, 500, 501, 502, 503, 504, 505, 4168, 4169, 506.4, 508.1, 508.8	J40~J47, J60~J67, I27.8, I27.9, J68.4, J70.1, J703	1
Rheumatic disease	725, 446.5, 710.0, 710.1, 710.2, 710.3, 710.4, 714.0, 714.1, 714.2, 714.8	M05, M06, M32, M33, M34, M31.5, M35.1, M35.3, M36.0	1
Peptic ulcer disease	531, 532, 533, 534	K25, K26, K27, K28	1
Mild liver disease	570, 571, 070.6, 070.9, 573.3, 573.4, 573.8, 573.9, V42.7, 070.22, 070.23, 070.32, 070.33, 070.44, 070.54	B18, K73, K74, K70.0, K70.1, K70.2, K70.3, K70.9, K71.3, K71.4, K71.5, K71.7, K76.0, K76/2, K76/3, K76.4, K76.8, K76.9, Z94.4	1
Diabetes without chronic complication	250.0, 250.1, 250.2, 250.3, 250.8, 250.9	E10.0, E10.l, E10.6, E10.8, E10.9, E11.0, E11.1, E11.6, E11.8, E11.9, E12.0, E12.1, E12.6, E12.8, E12.9, E13.0, E13.1, E13.6, E13.8, E13.9, E14.0, E14.1, E14.6, E14.8, E14.9	1
Diabetes with chronic complication	250.4, 250.5, 250.6, 250.7	E10.2, E10.3, E10.4, E10.5, E10.7, E11.2, E11.3, E114, E11.5, E11.7, E12.2, E12.3, E12.4, E12.5, E12.7, E13.2, E13.3, E13.4, E13.5, E13.7, E14.2, E14.3, E14.4, E145, E14.7	2
Paraplegia	342, 343, 3341, 344.0, 344.1, 344.2, 344.3, 344.4, 344.5, 344.6, 344.9	G81, G82, G04.1, G11.4, G80.1, G80.2, G83.0, G83.1, G83.2, G83.3, G83.4, G83.9	2
Renal disease	582, 585, 586, V56,	N18, N19, I12.0, I13.1,	2

	588.0, V42.0, V45.1, 403.01, 403.11, 403.91, 404.02, 404.03, 404.12, 404.13, 404.92, 404.93, 583.0, 583.1, 583.2, 583.3, 583.4, 583.5, 583.6, 583.7	N03.2, N03.3, N03.4, N03.5, N03.6, N03.7, N05.2, N05.3, N05.4, N05.5, N05.6, N05.7, N25.0, Z49.0, Z49.1, Z49.2, Z94.0, Z99.2	
Malignant cancer	140~172, 1740~1958, 200~208, 238.6	C43,C88, C00~C26, C30~C34, C37~C41, C45~C58, C60~C76, C81~C85, C90~C97	2
Severe liver disease	456.0, 456.1, 456.2, 572.2, 572.3, 572.4, 572.8	I85.0, I85.9, I86.4, I98.2, K70.4, K71.1, K72.1, K72.9, K76.5, K76.6, K76.7	3
Metastatic solid tumor	196, 197, 198, 199	C77, C78, C79, C80	6
HIV/AIDS	042, 043, 044	B20, B21, B22, B24	6

Table S3. Comparison of Kaplan–Meier estimate.

**Outcomes: in-hospital mortality (Measure: Hazard ratio)**			
Category 1	1.793 (1.159–2.775)	2.722 (1.742–4.253)	2.744 (1.744–4.315)
–	Category 2	1.518 (1.001–2.302)	1.530 (1.002–2.337)
–	–	Category 3	1.008 (0.653–1.555)
–	–	–	Category 4

Data are presented as the hazard ratio with 95% confidence interval in the column-defining category compared with row-defining category.

Comparisons should be read from left to right.

CI: confidence interval; HR: hazard ratio.

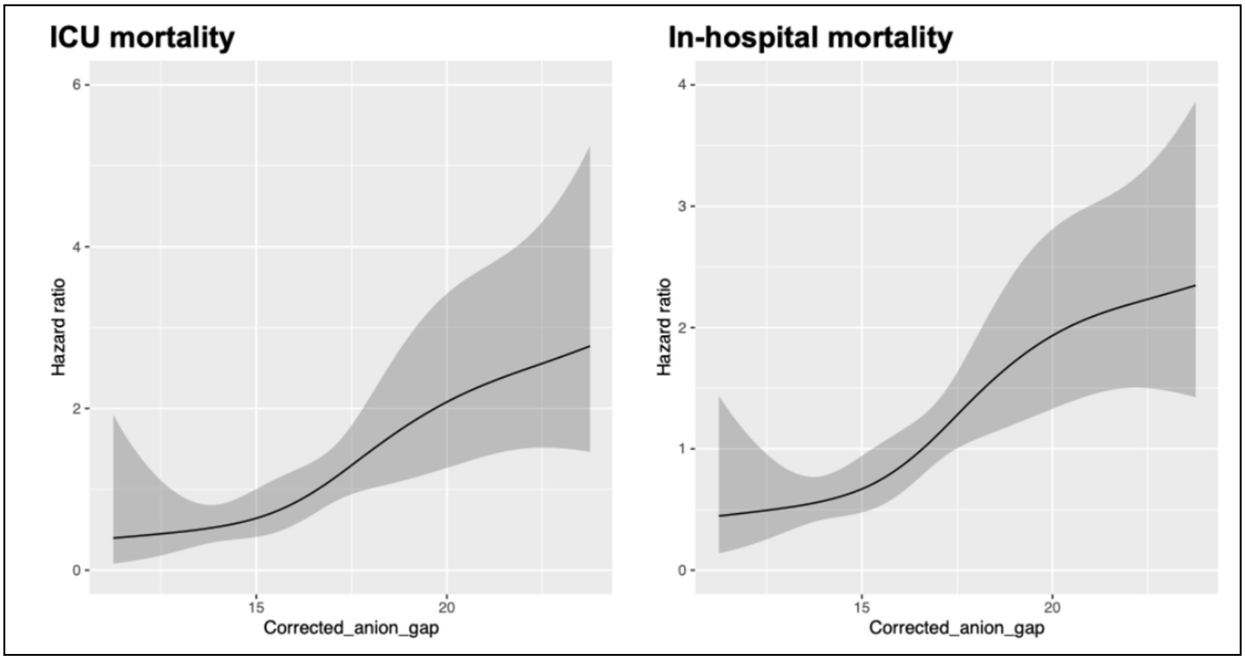

Figure S1. Relationship between the plasma corrected anion gap and the risk of intensive care unit and in-hospital mortality. Shaded areas around the curves depict 95% confidence intervals. ICU: intensive care unit.

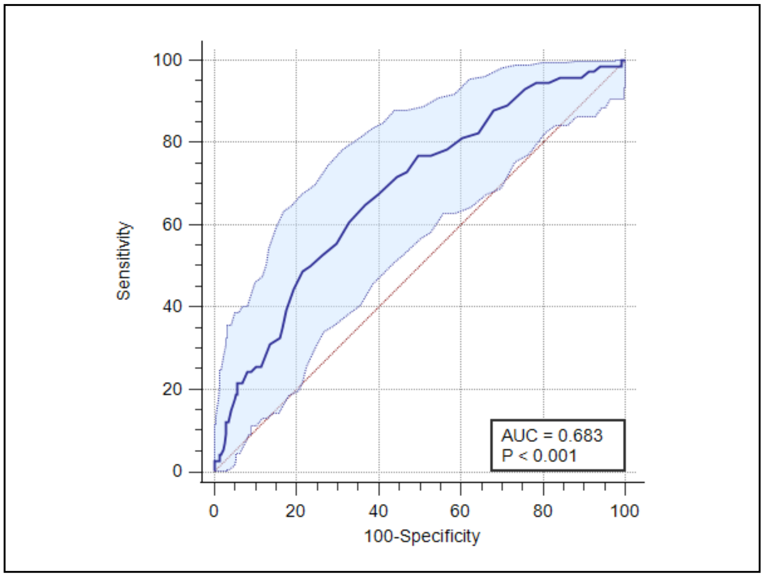

Figure S2. Receiver operating characteristic curves for the predictive ability of plasma corrected anion gap for the in-hospital mortality in stroke patients.
